# Weighted spectral clustering for water distribution network partitioning

**DOI:** 10.1007/s41109-017-0033-4

**Published:** 2017-06-30

**Authors:** Armando Di Nardo, Michele Di Natale, Carlo Giudicianni, Roberto Greco, Giovanni Francesco Santonastaso

**Affiliations:** 1 Dipartimento di Ingegneria Civile Design Edilizia e Ambiente, Università degli Studi della Campania ‘L. Vanvitelli’, via Roma 29, Aversa, 81031 Italy; 2 Action Group CTRL + SWAN of the European Innovation Partnership on Water EU, via Roma 29, Aversa, 81031 Italy; 3 Istituto Sistemi Complessi (Consiglio Nazionale delle Ricerche), Via dei Taurini 19, Rome, 00185 Italy

**Keywords:** Laplacian spectrum, Spectral clustering, k-means, Water network partitioning

## Abstract

In order to improve the management and to better locate water losses, Water Distribution Networks can be physically divided into District Meter Areas (DMAs), inserting hydraulic devices on proper pipes and thus simplifying the control of water budget and pressure regime. Traditionally, the water network division is based on empirical suggestions and on ‘trial and error’ approaches, checking results step by step through hydraulic simulation, and so making it very difficult to apply such approaches to large networks. Recently, some heuristic procedures, based on graph and network theory, have shown that it is possible to automatically identify optimal solutions in terms of number, shape and dimension of DMAs. In this paper, weighted spectral clustering methods have been used to define the optimal layout of districts in a real water distribution system, taking into account both geometric and hydraulic features, through weighted adjacency matrices. The obtained results confirm the feasibility of the use of spectral clustering to address the arduous problem of water supply network partitioning with an elegant mathematical approach compared to other heuristic procedures proposed in the literature. A comparison between different spectral clustering solutions has been carried out through topological and energy performance indices, in order to identify the optimal water network partitioning procedure.

## Introduction

Civil engineering networks regard different infrastructures (e.g. transport, energy, phone, internet, water, gas, logistic). Water Distribution Networks (WDNs) are among the most important civil networks, because they deliver drinking and industrial water to metropolitan areas. From a topological perspective, a WDN with multiple interconnected elements may be represented essentially as a link-node planar weighted spatially organized graph for which pipes (and valves) correspond to links *m*, and nodes/junctions (such as pipe intersections, water sources and nodal water demands) correspond to graph nodes *n*. Planar graph have vertices whenever two edges cross, whereas non-planar graphs can have edges crossing but not forming vertices (Boccaletti et al., 2006). WDN belong to the class of networks with nodes occupying precise positions in two or three-dimensional Euclidean space, edges being real physical connections, and strongly constrained by their geographical embedding (Boccaletti et al, 2006), like other spatially organized urban infrastructure systems (Carvalho et al, 2009; Newman, 2003).

In an abstract modelling context, a mathematical graph can be used to express the relationships between groups of linked nodes. An important aspect of spatial networks is that node degrees are constrained, as the number of possible connections to a single node is physically limited. Furthermore, in WDN it is unlikely to have direct connections between very distant nodes, so that significant limitations to the small-world behaviour of such networks arise (Boccaletti et al, 2006). In particular, little variability is observed in the connectivity patterns of the nodes in WDN, no hubs (nodes with much more connections than the others) are present, and most of the nodes have very low degree (usually two or three, and mostly less than five), so in general they present a fairly homogeneous degree distribution (Di Nardo et al, 2015a). Furthermore, such networks are also equally sensitive to random or malicious failures (Barthelemy and Flammini, 2008).

WDN can be considered as complex networks for many reasons (Mays, 2000): they are often very large (up to tens of thousands nodes and links); they are buried underground, and thus are not easily accessible for monitoring and maintenance; they are strongly looped; their modelling includes non-linear equations requiring sophisticated numerical resolution methods; they often present severe water losses. Compared to other civil networks (e.g. gas, electricity, transport, telephone, internet), some of these WDN characteristics are peculiar, and make their management arduous, with many operational problems (such as water and energy losses). For all these reasons, in the last decades, the scientific community has proposed different approaches to improve WDNs management, without compromising their main function, i.e. providing water to end users ensuring a minimum level of service.

In this context, the implementation of the paradigm of “divide and conquer” in a WDN allows simplifying the management, defining sub-systems named District Meter Areas (DMAs), by inserting gate valves and flow meters along network pipes, properly selected, in order to define a Water Network Partitioning (WNP). In this way, it is possible to improve water losses identification (Water Industry Research Ltd, 1999), control district pressure (Alonso et al, 2000), and protect users from accidental and intentional contamination (Di Nardo et al, 2015b), because these activities are simpler to achieve if the network is divided in sub-systems. By dividing the water network in DMAs, implementing innovative Information and Communications Technology (ICT) remote-controlled devices and big data analysis, it is possible to change the traditional approach to the management of WDN, transforming the water systems into modern Smart Water Network (SWAN) (Di Nardo et al, 2016a), considered as part of Smart Cities.

It is important to underline that, to define a good WNP, it is necessary to satisfy two crucial major requirements for the optimal functioning of a WDN: 1) network connectivity, i.e. each demand node of the water network must be connected to at least one water source, and 2) nodal minimum pressure, i.e. each node must have a pressure equal or higher than the minimum level of service that allows satisfying the water demand of the users. Therefore, the design of a WNP, as any problem of network subdivision, is a complex challenge for operators, because the permanent partitioning changes the original topological layout of water systems. Indeed, network partitioning, achieved by pipe closures, reduces the overall pipe section availability, with the consequent decrease of network water pressure, especially during peak hours, worsening the level of service offered to users.

In the last years, different procedures have been proposed in the literature for finding an optimal WNP layout (reviews are given in Di Nardo et al, 2013a; Perelman et al, 2015), essentially based on heuristic algorithms and optimization procedures. Generally, they consist of two different phases:

- **clustering**, aimed at defining the shape and the dimensions of the network subsets, based on different theories, among which: *graph theory algorithms*, obtaining the number of independent sectors through connectivity analysis, (Tzatchkov et al, 2006); identifying the pipes along which to insert hydraulic devices by searching minimum dissipated power paths using graph theory principles (Di Nardo et al, 2013a; Alvisi and Franchini, 2014); with an optimization model solved by a simulated annealing algorithm with an objective economic function (Gomes et al, 2012); based on shortest path search with dissipated power weight on pipes and refining through an objective function of the Genetic Algorithm based on network mean pressure (Di Nardo et al, 2013b); *spectral approach* with spectral clustering algorithm applied to adjacency matrix with different supply constraints (Herrera et al, 2010) or recursive bipartition of the graph through weighted graph Laplacian matrix (Di Nardo et al, 2017); *multi-agent approach* taking into account multiple interacting agents of WDN (Izquierdo et al, 2011); *community structure*, based on social network theory and graph partitioning algorithms (Di Nardo et al 2015a) or with an automatic identification of boundaries on the basis of the property that density of edges within communities should be higher than between them (Diao et al, 2013);
- **dividing**, aimed at physically partitioning the network, by selecting pipes for the insertion of flow meters or gate valves: based on *recursive bisection* procedure and an algorithm for graph traversal to verify the reachability of each district from the water source and node connectivity (Ferrari et al, 2014); on *genetic algorithms* implementing an automatic heuristic optimization technique for DMAs definition with minimum hydraulic deterioration (Di Nardo et al., 2015c, 2016b), with the objective of identifying the optimal layout that minimises the economic investment and the hydraulic performance deterioration.

Generally, such a two steps approach allows simplifying the water network partitioning, as, once the optimal node clustering is identified, then it becomes the starting point of the subsequent dividing phase. It is worth to highlight that the proposed procedures can be more effective if the clustering phase takes into account some hydraulic features of the network (i.e., energy, geometry), as reported in other studies (Di Nardo et al., 2013a, 2016a) depending on the adopted clustering algorithm. To such aim, in this work, the most important energy parameters are taken into account for the clustering stage.

This paper, extending a previous basic work (Di Nardo et al, 2017), aims at investigating the feasibility of adopting weighted spectral clustering to identify the optimal sub-graphs layout, comparing different weights of pipes and different spectral methods (von Luxburg, 2007), and then, subsequently, to define the optimal water network partitioning not only from a topological but also from a hydraulic point of view.

## Methodology

As described above for other approaches, the proposed procedure consists of two distinct phases (Di Nardo et al, 2016a), separately described in the following subsections.

## Phase 1: water network clustering

As known, considering a simple graph *G = (V,E)*, where *V* is the set of *n* vertices *v*_*i*_ (or nodes) and *E* is the set of *m* edges *e*_*l*_ (or links), a k-way graph clustering problem consists in partitioning *V* vertices of *G* into *k* subsets, *P*_*1*_ *, P*_*2*_ *,…, P*_*k*_ such that:$\bigcup_1^k{P}_k= V$ (the union of all clusters *P*_*k*_ must contain all the vertices *V*_*i*_), *P*_*k*_ *∩P*_*t*_ *= Ø* (each vertex can belong to only one cluster *P*_*i*_), *Ø ⊂ P*_*k*_ *⊂ V* (at least one vertex must belong to a cluster and no cluster can contain all vertices) and *1 < k < n* (the number *k* of clusters must be different from one and from the number *n* of vertices). Clustering is usually defined in terms of weighted, undirected graphs, where weights correspond to either similarity scores, or distances, or, more generally, they express the strength of the link between elements in order to define sub-graphs which take into account proximity and/or similarity between elements.

Graph clustering can be achieved with many procedures aimed to define the optimal layout of each cluster, finding community structures minimizing or maximizing an objective function that emphasizes one of the clustering aims. In literature (wide reviews are provided in Boccaletti et al, 2006; Fortunato, 2010), several procedures were proposed: k-means; Markov cluster algorithm; spectral methods (as optimization algorithm of the cut problem, such as min-cut, ratio-cut, normalized-cut); hierarchical clustering; modularity; multi-level-recursive algorithm, Girvan and Newman algorithm and some other methods.

In recent years, spectral clustering, based on eigenvectors and eigenvalues of the graph Laplacian matrices (defined hereinafter), has become one of the most popular clustering algorithms (Chung, 1997; Saerens et al., 2004; von Luxburg, 2007), because it can be solved by standard linear algebra software developed by the authors in MATLAB™ (SimuLink Reference Books 2006) and so it is easy to implement. So, in this paper, the clustering phase to define sub-graph for the subsequent dividing phase has been achieved with different weighted spectral clustering techniques, investigating the effectiveness of this approach and the optimal choice of weights. As known, the main tools for spectral clustering are graph Laplacian matrices and, in the following, *G* is assumed as an undirected, weighted graph with weight matrix *W*_*ω*_, where *w*_*ij*_ *= w*_*ji*_ *≥ 0*. In particular, as explained above, different weights have been adopted for the pipes to investigate which of them provides the best results. The choice of pipe weights is crucial, as different weights lead to significantly different layouts of the districts. As aim of the partitioning is to identify a balanced layout of the districts (i.e. districts with similar dimensions) least affecting the hydraulic performance of the network (i.e. minimising the unavoidable increase of head losses), pipe characteristics related to hydraulic resistance have been here tested as weights.

Given a graph *G = (V, E)*, the adjacency *nxn* matrix *A* (in the following indicated as *W*_*A*_ and corresponding to the no-weight matrix) expresses the connectivity of the graph, where elements *a*_*ij*_ *= a*_*ji*_ = 1 indicate that there is a link between nodes *i* and *j* and *a*_*ij*_ *= a*_*ji*_ = 0 otherwise.

Three spectral clustering methods have been tested. The first one, which solves relaxed versions of the RatioCut problem (von Luxburg, 2007), is based on the eigenvalues of the unnormalized graph Laplacian *L*, defined as:

1 $$ L={D}_k{\textstyle \hbox{-} }{W}_{\omega} $$

where *D*_*k*_ = diag(*K*_*i*_) and *K*_*i*_ is the degree of a node *i*.

The other two methods, both solving relaxed versions of the NCut problem (Shi and Malik, 2000), belong to normalized spectral clustering, as they use the eigenvalues of normalized graph Laplacian. In particular, the normalized spectral clustering according to Shi and Malik (2000) is based on the normalized Laplacian *L*_*rw*_, closely related to a random walk (von Luxburg, 2007) and defined as:

2 $$ {L}_{rw} = {D_k}^{-1} L $$

The third tested method is the normalized spectral clustering proposed by Ng et al. (2001), based on eigenvectors of the normalized Laplacian *L*_*sym*_, a symmetric matrix defined as:

3 $$ {L}_{sym} = {D_k}^{-1/2} L\ {D_k}^{-1/2} $$

The above mentioned three spectral clustering algorithms have been applied to identify the optimal clusters in a WDN. Namely, the tested *W*_*ω*_ matrices have been: *W*_*A*_ (i.e.no weights are given to the pipes, so to take into account only the connectivity); *W*_*D*_ (weight equal to pipe diameter *D*, related to pipe hydraulic resistance in formulas with exponent close to -5); *W*_*1/L*_ (weight equal to the inverse of pipe length, linearly related to pipe hydraulic resistance); *W*_*C*_ (weight equal to pipe conductance, here assumed as proportional to *D* ^5^/*L*, under the simplifying hypothesis that all the pipes in the network share the same roughness coefficient); *W*_*F*_ (weight equal to pipe flow, indirectly related to both pipe hydraulic conductance and water demand distribution at nodes).

Specifically, the clustering phase for the proposed water network partitioning consists of the following steps:

1. abstraction of the water supply network as a graph *G = (V, E);*
2. definition of adjacency matrix and pipe weight matrices *W*_*ω*_ as defined above;
3. computation of the spectrum of unnormalized Laplacian matrix based on adjacency matrix in order to define the best number of clusters, *k*, according to the *k*-smallest eigenvalue, as explained below;
4. computation of the first *k* eigenvectors of unnormalized and of two normalized Laplacian matrices for all weight matrices *W*_*ω*_;
5. definition, for all the weights and for the three spectral algorithms, of the matrix *U*_*nxk*_ containing the first *k* eigenvectors as columns;
6. clustering the nodes of the network into clusters *C*_*1*_ *,…,C*_*k*_ using the *k*-means algorithm applied to the rows of the *U*_*nxk*_ matrix;
7. check of the continuity of the obtained clusters *C*_*k*_;
8. definition of the set of edge-cuts (or boundary pipes) *N*_*ec*_.

The boundary pipes are links for which the start node and the end node belong to different clusters *C*_*k.*_

It is important to highlight that in all three algorithms, an important aspect is to change the representation of the nodes *n* from Euclidian space to points of the matrix *U*_*nxk,*_ that enhances the cluster-properties in the data, so that clusters can be trivially detected in the new representation, in particular, through the simple *k*-means clustering algorithm (Tibshirani et al., 2001; von Luxburg, 2007).

## Phase 2: water network dividing

Phase 1 provides the edge-cut between clusters, i.e. the set of *N*_*ec*_ boundary pipes along which gate valves or flow meters must be installed. First, the number *N*_*fm*_ of flow meters to be inserted in the network is chosen, so that the remaining boundary pipes *N*_*bv*_ *= (N*_*ec*_ *-N*_*fm*_ *)* are closed by inserting gate valves. In order to simplify the water budget computation, it is better to keep *N*_*fm*_ as low as possible (Di Nardo et al, 2016b). This problem can be assimilated to a valve placement problem in WDNs. This is a NP-hard problem (Bodlaender et al., 2010) and it requires heuristic algorithms to find optimal solutions (Tindell et al., 1992). In other terms, once defined all the *e*_*ij*_ boundary pipes between clusters, those that must be closed must be chosen among all the possible combinations *N*_*DL*_ of water network partitioning layouts, expressed by the binomial coefficient:

4 $$ {N}_{DL}=\left(\begin{array}{c}\hfill {N}_{ec}\hfill \\ {}\hfill {N}_{fm}\hfill \end{array}\right) $$

It is important to underline that *N*_*DL*_ can be, already for a small water supply network and for a small number *k* of DMAs, such a huge number that it is often computationally impossible to investigate all the solution space.

However, closing pipes to divide the districts significantly changes the network layout, reducing the topological connectivity and the energy redundancy and, consequently, worsening the hydraulic performance.

Therefore, an optimization technique has been developed, in order to find, once fixed the number of flow meters *N*_*fm*_, the optimal choice of the boundary pipes along which gate valves are to be inserted, by minimizing the alteration of the hydraulic performance and of the level of service for the users. This aim has been achieved by a heuristic procedure carried out with a Genetic Algorithm (*GA*) developed by the authors (Di Nardo et al, 2016a), maximizing the following objective function:

5 $$ \max \left(\gamma {\displaystyle \sum_{i=1}^n\left({z}_i+{h}_i\right)\cdot {Q}_i}\right) $$

corresponding to the total nodal power of the network (Di Nardo et al., 2013a), in which *γ* is the specific weight of water, *z*_*i*_, *h*_*i*_ and *Q*_*i*_ are, respectively, the geodetic elevation, the pressure and the water demand at the *i*-th node. The *GA* parameters are the following: each individual of the population is a sequence of *N*_*ec*_ binary chromosomes corresponding to the pipes belonging to the edge-cut set; the *l*-th chromosome is set to 1 if a gate valve is inserted along the corresponding *l*-th pipe, while it is set to 0 if a flow meter is inserted. The *GA* has been carried out with 100 generations and with a population consisting of 500 individuals with a crossover percentage equal to *P*_*cross*_ = 0.8.

In order to compute the objective function, hydraulic simulations are carried out in the GA. They are carried out using the freeware software EPANET2 (Rossman, 2000), that numerically solves the non-linear hydraulic equations of the water system.

Finally, after the dividing phase, hydraulic simulations are required to compute some performance indices (Di Nardo et al., 2015c) aimed at evaluating the hydraulic performance of WNP and so allowing to compare different layouts.

## Case study

The effectiveness of the proposed procedure has been tested for the real case study of the water supply network of Parete, a town with 10,800 inhabitants, located in a densely populated area near Caserta (Italy). The water network has two sources and its main topological and energy characteristics are reported in Tables 1 and 2, respectively.

**Table 1:** Topological characteristics of the water distribution network of Parete

m	n	q	K	APL	Dm	λ_2_	Δλ
282	184	0.017	3.05	8.80	20	0.021	0.062

**Table 2:** Hydraulic characteristics of the water distribution network of Parete

h*	h_min_	h_mean_	h_max_	P_A_
[m]	[m]	[m]	[m]	[kW]
25.00	21.36	31.05	50.47	12122.11

h* = 25 m (the pressure head required to satisfy water demand at all nodes)

The network consists of *m* = 282 links and *n* = 184 nodes and, from a topological point of view, in agreement with most real systems, it is a sparse network, so it is not fully connected and its number of edges *m* < <*n* ^2^, with a link density value (i.e. the ratio between the actual number of links and the number of links of a fully connected network with the same number of nodes) *q* = 0.017. As the number of edges that can be connected to a single node is limited by the physical space in spatial networks (Boccaletti et al, 2006), average node degree $\widehat{K}$=3.05 is small. The case study shows a small average path length *APL* = 8.80, presenting itself as a cohesive and robust network (Yazdani and Jeffrey, 2011) as well as the value of graph diameter *Dm = 20* shows that the nodes are mutually and easily reachable and that the network is ordered in a decentralized fashion (Yazdani and Jeffrey, 2010), which is an important aspect for an efficient communication (the flow in the case of hydraulic networks). Concerning the main spectral measurements, the “spectral gap” *Δλ* (the difference between the two largest eigenvalues of the adjacency matrix) is equal to 0.062 and the “algebraic connectivity” *λ*_*2*_ (Fiedler, 1973) (the second smallest eigenvalue of the Laplacian matrix) is equal to 0.021. Both these values are small, showing that the graph arrangement can be decomposed into isolated parts (clusters or districts) (Estrada, 2006).

The hydraulic performances of the water supply network of Parete, reported in Table 2, is good in terms of maximum and mean nodal pressure heads, with *h*_*max*_ and *h*_*mean*_ higher than the design pressure head *h* ^*^ = 25 m (the pressure head required to satisfy water demand at all nodes). Conversely, the minimum pressure head *h*_*min*_ is lower than *h* ^*^, indicating that in some nodes the design pressure requirement is not fulfilled. Consequently, the system shows little energy resilience and so a “low availability” of the water system to be partitioned without a decrease in hydraulic performance (Greco et al, 2012). In Table 2 the value of the input power P_A_ (a global performance index measuring the amount of energy entering the water system through the reservoirs and provided by pumps) is also reported.

**Fig. 1:**
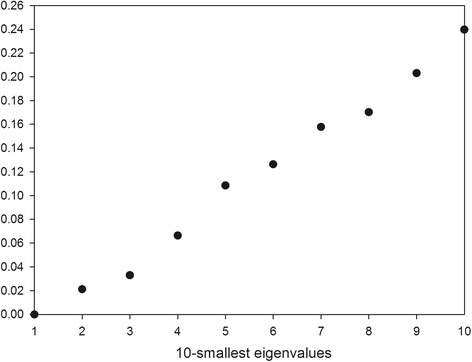
First *10*-smallest eigenvalues of unweighted graph Laplacian matrix

Following the steps of the proposed methodology for water network partitioning, WDN first can be seen as a graph (step 1) *G = (V, E)* in which *V* is the set of *n* vertices *v*_*i*_ (the junction, the delivering nodes and the reservoirs) and *E* is the set of *m* edges *e*_*l*_ (the pipes connecting the nodes). Then, all the above defined five weight matrices *W*_*ω*_ are computed (step 2) and used to choose the most appropriate number *k* of clusters; this is a common problem in all clustering algorithms. The tool designed for spectral clustering, the eigengap heuristic (von Luxburg, 2007), is applied to all three graph Laplacian matrices, choosing the number of clusters *k* such that all eigenvalues *λ*_*1*_,…,*λ*_*k*_ assume small and similar values, while *λ*_*k+1*_ is relatively larger (step 3). According to this criterion, as shown in Fig. 1 for the case study of the water network of Parete, relatively to no-weight Laplacian matrix, where the first 10 smallest eigenvalues are plotted, the most appropriate number of clusters is three or four. It is worth to note that, as explained in von Luxburg, (2007), the eigengap heuristic works well only if the clusters in the data are very well pronounced, i.e. the more overlapping the clusters are, the less clear is the detection of the number of clusters. However, such a method gives in any case a useful preliminary indication.

**Fig. 2:**
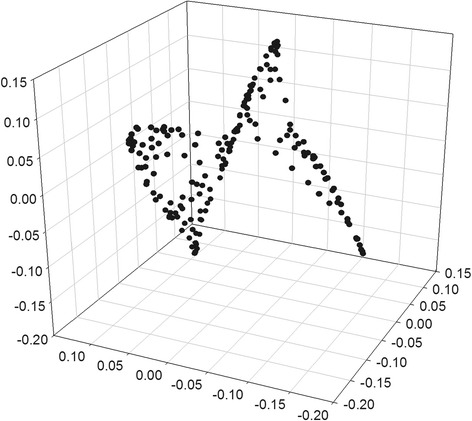
Node coordinates in the eigenspace of the first *3*-smallest eigenvectors of the diameter-weighted graph *L*_*rw*_ Laplacian matrix

Once fixed the number *k* = 4 of clusters into which the network is subdivided, the first phase of the proposed partitioning procedure provides the spectral clustering of the water supply network of Parete. A total number of clustering layouts *N*_*CL*_ = 15 is obtained (three algorithms for five weight matrices), as reported in Table 3. The result of the partitioning of the graph is represented in Fig. 2, without loss of generality, for the case of pipe diameter as weight and *L*_*rw*_ Laplacian matrix. The network nodes are plotted in the eigenspace of the first three non-constant eigenvectors. The division into four sub-regions is evident, as the points result clearly arranged into four distinct groups. The results in terms of topological metrics are reported, for each Laplacian matrix and for each weight combination in Table 3, which gives: the number of nodes *n*_*k*_ of each cluster, the balanced node index *I*_*b*_ (standard deviation of the total number of nodes of the four clusters), the number *N*_*ec*_ of pipes of the edge-cut set.

**Table 3:** Characteristics of the clusters obtained with the three tested algorithms and the five adopted pipe weights

Laplacian Matrix	Weight	n° nodes DMA 1	n° nodes DMA 2	n° nodes DMA 3	n° nodes DMA 4	I_b_	N_ec_
L	W_A_	48	48	43	45	2.45	16
	W_D_	53	46	43	42	4.97	17
	W_1/L_	59	56	40	29	14.07	20
	W_C_	84	65	19	16	33.83	26
	W_F_	76	64	42	2	32.54	15
L_rw_	W_A_	48	48	45	43	2.45	16
	W_D_	49	47	45	43	2.58	17
	W_1/L_	54	45	44	41	5.60	19
	W_C_	82	40	33	29	24.43	21
	W_F_	78	43	36	27	22.32	24
L_sym_	W_A_	48	48	45	43	2.45	16
	W_D_	49	47	45	43	2.58	17
	W_1/L_	55	47	42	40	6.68	19
	W_C_	126	32	15	11	54.10	-
	W_F_	86	46	28	24	28.33	-

For the two last solutions in Table 3 (*W*_*C*_ and *W*_*F*_ weight matrices with *L*_*sym*_ as Laplacian matrix), the continuity of the network is not ensured. For the continuity check, it has been exploited another important property of the Laplacian matrix, namely the multiplicity *m*_*a*_ of its zero eigenvalue, that is equal to the number of connected sub-graphs of the network. So, the multiplicity of the zero eigenvalue of the unweighted unnormalized Laplacian matrix of the graph subdivided into four clusters has been evaluated. It resulted *m*_*a*_ = 4, indicating that the obtained sub-graphs result internally connected, while, if *m*_*a*_ > 4, it would mean that the network has been divided into more than four sub-graphs.

It is also evident that the most balanced layouts (i.e. clusters with similar numbers of nodes) correspond to the *W*_*A,*_ *W*_*D*_ and *W*_*1/L*_ for all three Laplacian matrices: as reported in Table 3, they lead to the lowest values of *I*_*b*_. As expected, the most balanced layout corresponds to no-weight matrix. In fact, without any weight, the spectral clustering leads to sub-graphs containing similar numbers of links that, in a WDN like Parete, implies also similar numbers of nodes (indeed the number of links connected to a node varies only slightly throughout the network). Conversely, when weights are given to pipes, the sum of the weights of the pipes belonging to the clusters is balanced, which does not necessarily imply that the clusters contain similar numbers of nodes. The last step of the clustering phase is the definition of the edge-cut set. This phase must be achieved with the aim of minimizing, in the subsequent dividing phase, the network perturbation and the investment related to the insertion of hydraulic devices. In this respect, it is reasonable that edge-cut sets containing a small number *N*_*ec*_ of intra-cluster pipes would be preferable. Also for this index, the optimal solutions correspond to *W*_*A,*_ *W*_*D*_ and *W*_*1/L*_ for all three Laplacian matrices (they lead to the lowest values of *N*_*ec*_). Even if the *W*_*F*_ with *L* Laplacian matrix provides the lowest *N*_*ec*_, such a solution should not be considered, as it corresponds to a very unbalanced cluster layout.

The results given in Table 3 should be interpreted considering that, while unweighted spectral clustering provides the edge-cut set with the minimum *N*_*ec*_ (compatible with the requirement of obtaining balanced clusters), weighted spectral clustering minimises the sum of the weights of the edges constituting the edge-cut set. Hence, it was expected that such a minimization would have not necessarily led to small values of *N*_*ec*_.

In this respect, it is interesting to note that the application of weights resulted in edge-cut sets less suitable to carry out the following dividing phase (achieved with EPANET software embedded in the GA) with little disturbance to the hydraulic performance of the network, if compared to the edge-cut set identified by the unweighted spectral clustering. In fact, for the dividing phase to affect as little as possible the hydraulic performance, the closed pipes should be the ones carrying small flows (pipes with small conductance, i.e. with small diameter), while the pipes remaining open after the dividing phase (i.e. the pipes along which water meters are installed) should be large, as they have to be capable of carrying also the discharge which, before the division, flew through the closed pipes. In other words, the edge-cut set should contain pipes with both small and large diameters (or hydraulic conductance). As reported in Table 4, which gives the diameters of the pipes belonging to the edge-cut sets obtained with different weights, in the case of the WDN of Parete, the edge-cut set provided by the unweighted clustering has such a feature. Conversely, the more the adopted weights are effective (i.e. the weights assume very different values for the various pipes of the network), the more the pipes of the edge-cut tend to be homogeneous (for instance, when the hydraulic conductance is adopted as a weight, the edge-cut set is formed by only very small pipes).

**Table 4:** Characteristics of the edge-cut set obtained with the three tested algorithms and the five assumed pipe weights

Laplacian Matrix	Weight	N_ec_	Multiplicity of pipe diameters D [mm]						
			60	80	100	110	125	150	200
L	W_A_	16	5	1	4	1	1	2	2
	W_D_	17	6	1	4	1	1	2	2
	W_1/L_	20	5	1	3	0	1	3	7
	W_C_	26	20	0	2	1	1	1	1
	W_F_	15	4	2	4	0	1	1	3
L_rw_	W_A_	16	5	1	4	1	1	2	2
	W_D_	17	6	1	4	1	1	2	2
	W_1/L_	19	7	1	3	1	1	2	4
	W_C_	21	10	3	3	1	1	3	0
	W_F_	24	10	1	4	1	1	2	6
L_sym_	W_A_	16	5	1	4	1	1	2	2
	W_D_	17	6	1	4	1	1	2	2
	W_1/L_	19	6	1	4	1	1	2	4
	W_C_	-	-	-	-	-	-	-	-
	W_F_	-	-	-	-	-	-	-	-

As shown in Table 5, which gives the main hydraulic performance indices of the network after the dividing phase (evaluated through the EPANET software), the number of flow meters has been fixed for all combinations to *N*_*fm*_ = 5, which is the minimum possible number that guarantees the hydraulic performance of the network and, at the same time, simplifies the computation of the water budget (Water Industry Research Ltd, 1999), allowing an easier identification of water losses. Clearly, the number of gate valves is in all cases equal to the difference *N*_*bv*_ = *N*_*ec*_-*N*_*fm*_.

**Table 5:** Hydraulic performance indices after the dividing phase for the three tested algorithms and the five adopted pipe weights

Laplacian Matrix	Weight	N_ec_	N_bv_	N_fm_	P_D_	P_N_	h_min_	h_mean_	h_max_
		[-]	[-]	[-]	[kW]	[kW]	[m]	[m]	[m]
L	W_A_	16	11	5	1831.64	10290.47	22.78	30.46	50.07
	W_D_	17	12	5	1875.29	10246.82	22.09	29.82	50.16
	W_1/L_	20	15	5	1890.23	10231.88	22.23	29.93	49.87
	W_C_	26	21	5	1954.84	10167.27	14.04	28.68	50.03
	W_F_	15	10	5	1716.15	10405.96	21.58	30.99	50.41
L_rw_	W_A_	16	11	5	1831.64	10290.47	22.78	30.46	50.07
	W_D_	17	12	5	1875.29	10246.82	22.09	29.82	50.16
	W_1/L_	19	14	5	1863.96	10258.15	22.49	30.24	49.98
	W_C_	21	16	5	1895.35	10226.76	20.81	29.13	50.61
	W_F_	24	19	5	1720.86	10401.25	20.79	30.81	50.50
L_sym_	W_A_	16	11	5	1831.64	10290.47	22.78	30.46	50.07
	W_D_	17	12	5	1875.29	10246.82	22.09	29.82	50.16
	W_1/L_	19	14	5	1877.49	10244.62	22.36	30.09	50.11
	W_C_	-	-	-	-	-	-	-	-
	W_F_	-	-	-	-	-	-	-	-

After the first clustering phase, the dividing phase has been carried out, computing all the hydraulic performance metrics reported in Table 5, namely: the dissipated power *P*_*D*_; the total nodal delivered power *P*_*N*_ *= P*_*A*_ *-P*_*D*_ (Di Nardo et al., 2013a); the minimum, mean and maximum pressure *h*_*min*_, *h*_*mean*_ and *h*_*max*_.

Obviously, the results for the two last clustering solutions (*W*_*C*_ and *W*_*F*_ weight matrices with *L*_*sym*_ Laplacian matrix) are not reported, because the continuity of the network is not respected and so the hydraulic simulation needed for the evaluation of the hydraulic performance could not be carried out.

It is important to highlight that the reported results are the optimal for each weight-Laplacian combination, meaning that, for the fixed number of flow-meters *N*_*fm*_ = 5 and within the investigated solution space, the power *P*_*D*_ dissipated by the system is minimized, and, consequently, the total nodal power *P*_*N*_ is maximized.

As expected, the results in terms of hydraulic performance indicate that the best solutions correspond to the *W*_*A,*_ *W*_*D*_ and *W*_*1/L*_ for all three Laplacian matrices, as they lead to the smallest numbers of closed pipes. Also in this phase, even if the clusters layouts obtained with *W*_*F*_ by means of both *L* and *L*_*rw*_ Laplacian matrix seem to correspond to the lowest value of dissipated power *P*_*D*_, they cannot be considered as good solutions, because they are very unbalanced.

For the presented case study, it is clear that, from both topological and hydraulic point of views, the best solutions have been achieved with the normalized *L*_*rw*_ Laplacian matrix. Indeed, at the same time it provides the most balanced clusters solutions, an edge-cut set with few pipes (Table 3), the lowest dissipated power, and the highest minimum pressure head. With reference to the weight choice, it looks clear that, even if there is not a great difference between *W*_*A,*_ *W*_*D*_ and *W*_*1/L*_, the best solution was achieved with the unweighted matrix, regardless of the adopted Laplacian matrix. In this respect, it is worth to note that this result cannot be generalized, as it depends on the peculiar distribution of pipe diameters of the analysed WDN. In fact, unweighted clustering takes into account only the topological structure of the network, without using any information related to the hydraulic characteristics of the pipes. However, the obtained results are good in terms of nodal pressure and confirm the suitability of spectral clustering for water network partitioning. Further investigation about the choice of the weights is required to define a spectral clustering approach of general validity for the definition of DMAs.

**Fig. 3:**
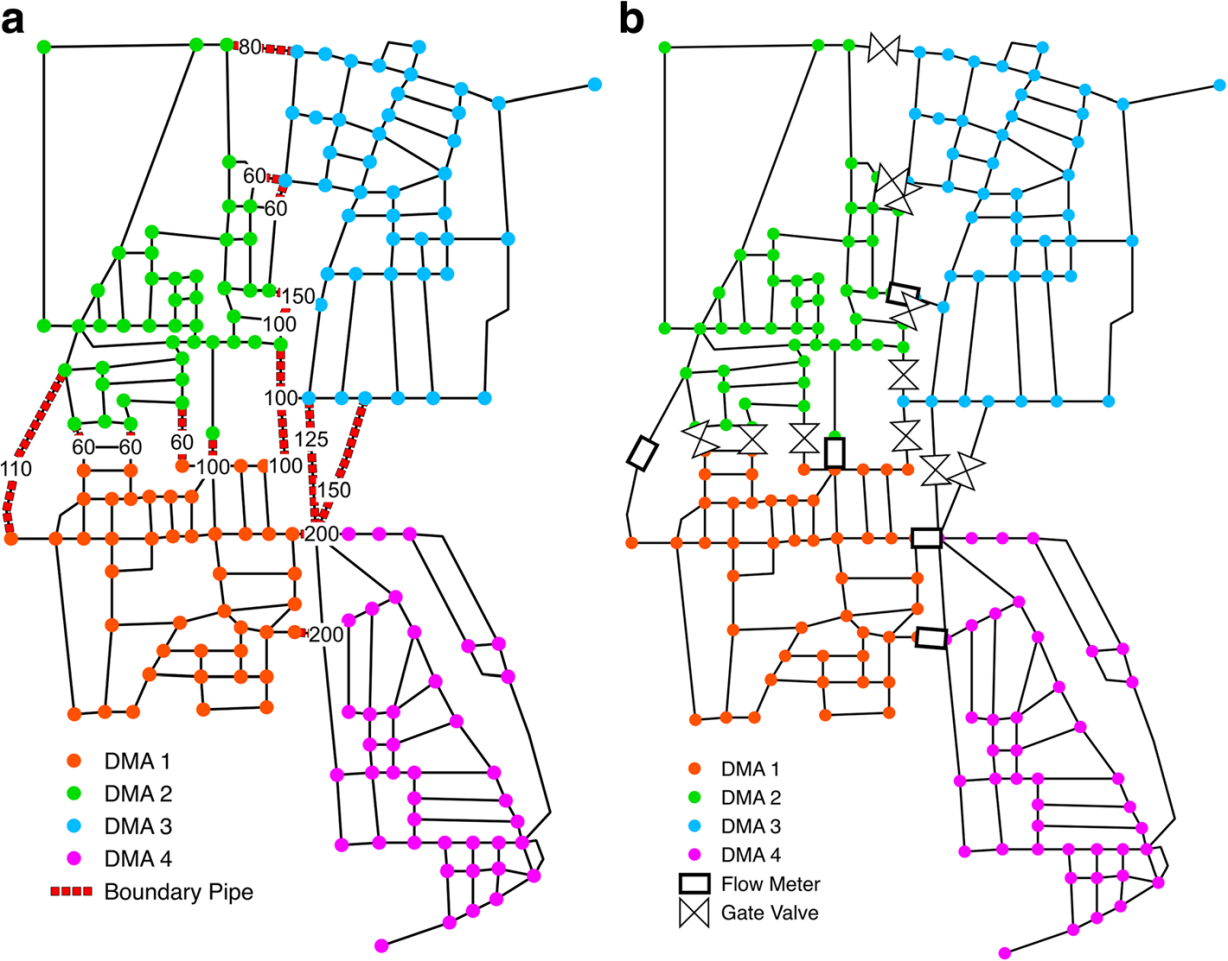
Parete WSN partitioning in *4*-DMA for the unweighted graph adjacency matrix: clustering phase (**a**) and dividing phase (**b**)

**Fig. 4:**
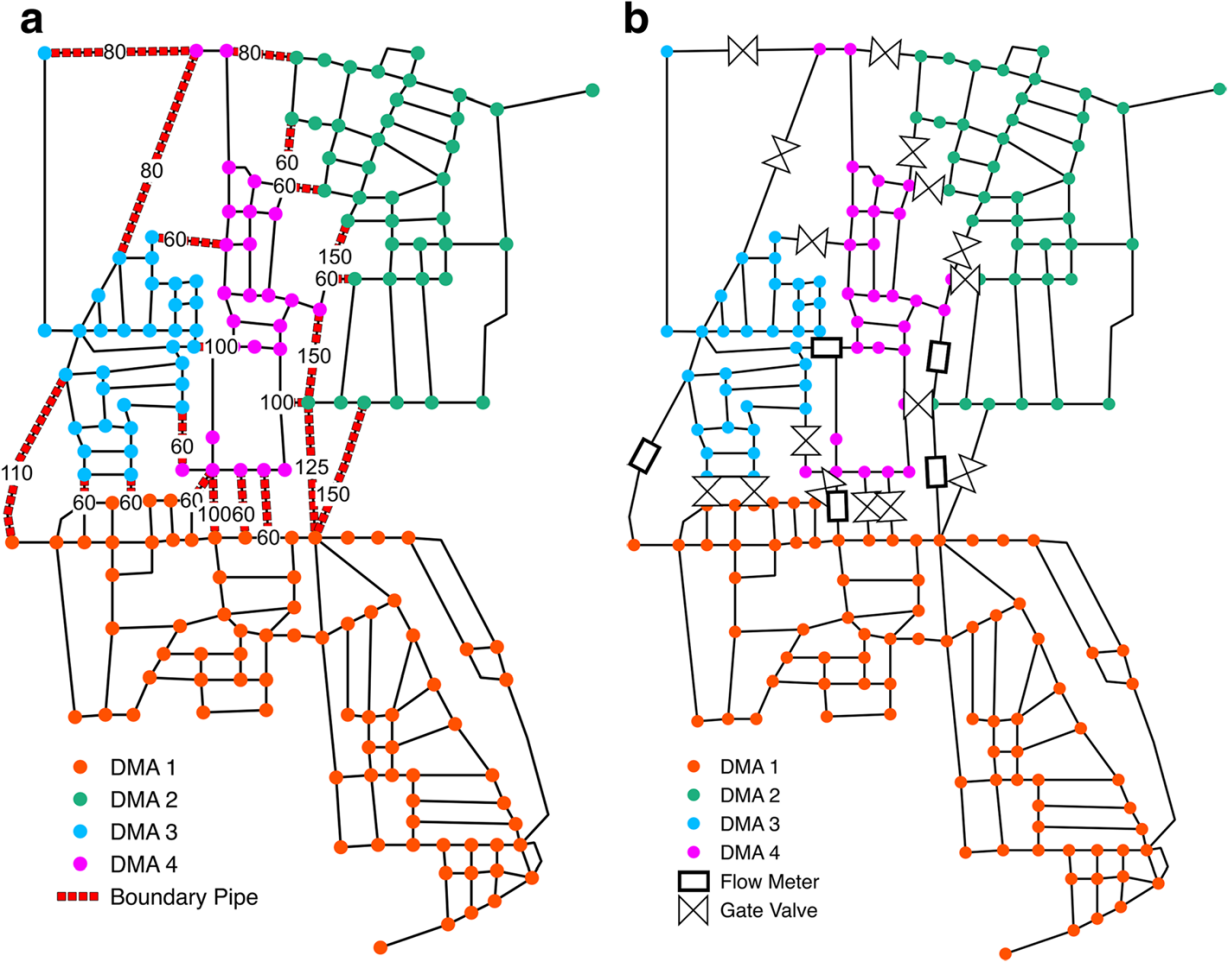
Parete WSN partitioning in *4*-DMA for the conductance-weighted graph adjacency matrix and *L*_*rw*_ Laplacian: clustering phase (**a**) and dividing phase (**b**). Flow meters are represented by rectangles while gate valves with the double triangle

Finally, Fig. 3 shows the WNP of the network of Parete corresponding to the best solution in terms of minimum pressure *h*_*min*_ = 22.78, obtained with unweighted matrix and *L*_*rw*_ Laplacian. In particular, in the left pane of Fig. 3, the first clustering phase is reported, highlighting the edge-cut set (dashed lines). In the right pane, the second dividing phase is illustrated, highlighting the optimal positioning of devices, which ensures the minimum hydraulic performance deterioration. For comparison, in Fig. 4, the WNP of Parete obtained with conductance-weighted adjacency matrix and *L*_*rw*_ Laplacian is reported, highlighting the less balanced obtained layout. The different shapes and dimensions of the obtained clusters, as well as the greater number of edge-cuts are also evident. In both cases (unweighted WNP and conductance-weighted WNP), the gate-valves (closing pipes) have been located along the pipes with smallest diameters by means of the *GA* algorithm, so to reduce hydraulic performance deterioration.

## Conclusions

The division of a WDN into DMAs aims at improving water supply network management and so, consequently, leakage detection and system safety. At the same time, the closure of pipes with gate valves to define the DMAs unavoidably increases the hydraulic head losses, leading to lower pressure at the water delivery nodes, compared to non-clustered layout. So far, although the design of optimal DMA layout is a problem deeply studied in the scientific literature, there is not an established procedure to solve it. In this regard, the paper presents an application to a real WDN of weighted spectral clustering for water network partitioning.

Spectral clustering is based on the eigenvalues of the graph Laplacian matrix of the network, for which three different formulations have been tested. Five different weights have been adopted, chosen among the major characteristics of the pipes: adjacency (in this case the partitioning is based only on the topology of the network), diameter, length, conductance and flow. Aim of the application is to understand which of the considered characteristics provides the best clustering layout, in terms of minimizing the edge-cuts and simultaneously balancing the dimensions of the clusters.

Compared to other heuristic methodologies, weighted spectral clustering allows to take into account either topological, geometrical, or hydraulic information about the system, within the framework of an elegant mathematical formalism.

Simulation results for the analysed case study, carried out with a number of DMAs *k* = 4, defined through the analysis of the eigenvalues of the unweighted Lapalcian matrix, confirm the effectiveness of the procedure, providing balanced clustering layouts and small numbers of intra-cluster boundary pipes. The latter result may favour the following heuristic dividing phase, consisting in the choice of the positions of flow meters and gate valves along the pipes of the edge-cut. Indeed, the hydraulic performance of the network, measured with several indices, is satisfactorily preserved in most of the weight-Laplacian combinations.

In particular, in this study the best solution was found, with the spectral clustering algorithm, using unweighted matrices. This result is different from previous studies found in the literature, in which different clustering techniques were adopted, and weighted matrices provided the best results. This result directly depends on the distribution of pipe diameters within the considered network, and therefore cannot be considered of general validity. In fact, it can be ascribed to the fact that, when weights related to pipe geometry are minimized, the optimal edge-cut tends to be formed by pipes with similar characteristics. In the dividing phase, instead, the best hydraulic results are obtained by installing gate valves along pipes with small diameter, and water meters along pipes with large diameter. Therefore, further investigation is required to define a weighted spectral clustering approach of general validity for the definition of DMAs.
